# Evaluating the feasibility of a remote-based training program supported by information and communications technology in the older adults living at home

**DOI:** 10.1186/s12877-022-03273-3

**Published:** 2022-07-13

**Authors:** Koji Oba, Yusuke Kagiwada, Masamitsu Kamada, Ryusuke Miki, Yuta Kondo, Tadashi Kamakura, Takeshi Yamagami, Tomomi Fujita, Yasuhiro Tsuchida

**Affiliations:** 1 Department of Biostatistics, Graduate School of Medicine, the University of Tokyo, Tokyo, Japan; 2 Interfaculty Initiative in Information Studies, the University of Tokyo, Tokyo, Japan; 3 Department of Health Education and Health Sociology, School of Public Health, Graduate School of Medicine, The University of Tokyo, Tokyo, Japan; 4 Health Planning Division, Public Health Bureau, Kobe City Government, Kobe, Japan; 5 Moff Corporation, Tokyo, Japan

**Keywords:** Elderly at home, Frail prevention, Remote intervention, Motion sensor

## Abstract

**Background:**

Exercise has been one of the key strategies for preventing frailty. While training programs for preventing frailty have been mainly developed in person, which have now become difficult to perform due to the coronavirus disease pandemic. It would be worthwhile to explore a feasibility of methods for a remote-based training with information and communications technology (ICT) in the pre-frail/robust older adults living at home.

**Methods:**

We assessed the feasibility of a remote-based training with ICT device in terms of 1) a measurement accuracy and 2) whether it could be used for remote-based training of different intensities. To evaluate a measurement accuracy of the ICT device, we evaluated an inter-rater reliability between a true score and scores obtaining from the ICT device in 20 participants aged 65 years and older. Intraclass correlation was calculated. To evaluate a feasibility of remote-based training interventions of different intensities, we did a parallel, randomized, active controlled trial. Participants aged 65 years or older were randomly allocated to the two 3-month intervention programs with different intensity of exercise with the ICT (i.e., an Exercise-Intensive program and a Light-load exercise program). The primary outcome was 3-month scores of the 30-s chair-stand test (CS-30), which was compared between two groups using mixed models for repeated measures to account for within-person correlations.

**Results:**

The ICT device showed a high intraclass correlation of over 0.99 for all outcomes including CS-30. Between Aug and Oct 2020, 70 participants (36 and 34 in the Exercise-Intensive and Light-load exercise programs, respectively) were randomized. After 3 months of intervention, CS-30 scores and other physical function improved in both groups. Difference in the 3-month CS-30 scores between two programs was found to be 0.08 (95% confidence interval: − 2.64, 2.79; *p* = 0.955), which was not statistically significant. No harmful incidents, such as falls, occurred in either group.

**Conclusion:**

We showed a remote-based training with ICT device in the older adults living at home was feasible. Further studies are warranted to determine what kind of remote exercise intervention programs is more effective for maintaining a physical performance and, beyond that, preventing frailty.

**Trial registration number:**

UMIN000041616 (05/09/2020) https://center6.umin.ac.jp/cgi-open-bin/ctr/ctr.cgi?function=brows&action=brows&recptno=R000047504&type=summary&language=E

## Background

Frailty is defined as “a clinically recognizable state of increased vulnerability resulting from an aging-associated decline in reserve and function across multiple physiologic systems such that the ability to cope with every day or acute stressors is compromised [1].” This condition arises from the weakening of homeostatic mechanisms that maintain the balance of multiple interrelated physiological systems [2, 3]. Considering that frailty is the third most common reason for long-term care [4], its prevention would contribute toward reducing the number of people needing long-term care/support. Studies have shown that the frailty prevalence rate increases with age [5], suggesting the importance of considering early prevention methods.

Conceptually, frailty can be divided into three domains: physical, psychological, and social [6]. Among these domains, physical frailty can be prevented or treated with specific modalities, such as exercise, protein-calorie supplementation, vitamin D supplementation, and polypharmacy reduction [7]. At present, exercise interventions are considered the most effective method for preventing the physical frailty, with studies recommending a multicomponent training regimen comprising resistance training, aerobic exercise, and balance and flexibility exercises [8–13]. Thus far, these training programs have been mainly conducted in person with supervision, which have now become difficult to perform due to the coronavirus disease (COVID-19) pandemic. Even in everyday life, reduced physical activity has apparently become a problem that leads to long-term care [14]. One of the solutions to address this problem has been the use of a remote-based training program supported by information and communications technology (ICT). In fact, a randomized controlled study by Dekker-van Weering et al. involving a 12-week exercise intervention for pre-frail older adults living at home that used ICT showed that such a program was simple to use and had the potential to improve health and quality of life [15]. However, while there have been clinical studies using ICT for different purposes, most of them, including the study by Dekker-van Weering et al., have focused on usability [16, 17]. Thus, investigating whether a training program using ICT can be implemented and would be as effective and safe as a program conducted in person would be worthwhile.

This current study evaluated the feasibility of a remote-based training program in terms of 1) a measurement accuracy of ICT device and 2) whether it could be used for remote-based training of different intensities. To evaluate a measurement accuracy of the ICT device, we evaluated an inter-rater reliability between a true score of outcomes which used in a remote-based training system and scores obtaining from the ICT device in individuals aged 65 years and older. To evaluate a feasibility of remote-based training interventions of different intensities, we conducted a randomized parallel-group open-label trial, the “PEERS trial” (PEERS: **P**revention of frailty in the **E**lderly living at home using **E**xercise with **R**emote **S**upervision), which was conducted using an ICT-based training system to evaluate the comparative effectiveness and safety of two remotely delivered frailty prevention exercise programs with different exercise doses on pre-frail/robust community-dwelling older adults Japanese individuals aged 65 years or older.

## Methods

This study consists of two parts. Since the inter-rater reliability of ICT device was not scientifically obtained, we evaluated the ICT device to confirm how accurately and reliably it could measure the outcomes used in the PEERS trial before the trial started (the section of “Inter-rater reliability of the ICT device”). After confirming the inter-rater reliability of the device, we conducted the PEERS trial (the section of “Prevention of frailty in the Elderly living at home using Exercise with Remote Supervision: the PEERS trial”). These studies were conducted jointly by the University of Tokyo, Moff Co., Ltd., and the Kobe City Government with the approval of the Ethics Committee of the Graduate School of Interdisciplinary Information Studies, University of Tokyo (approval no. 20–05). Moff Co., Ltd., is a for-profit company that provides services using this ICT device, and in this regard, we declare in advance that a conflict of interest exists for this study. All procedures were performed according to the ethical standards of the responsible committee on human experimentation (institutional and national) and the Helsinki Declaration of 1975, as revised in 2013. Written informed consent was obtained when the patients voluntarily agreed to participate in this study after explaining the purpose and procedures of this study.

## Inter-rater reliability of the ICT device

### Study design

**Fig. 1:**
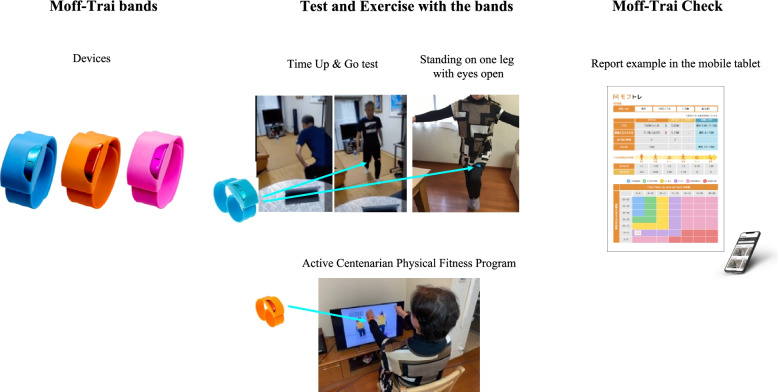
Overview of ICT-based training system named “Moff-Trai”. When participants exercise, they wear the Moff-Trai band. Bluetooth connection was established between the Moff-Trai band and the mobile tablet. Test scores or exercise time are sent to the application and participants can check the report on the mobile tablet

The Moff Corporation (Tokyo, Japan) had developed an ICT-based training system named “Moff-Trai” (short for “Moff Training”), which consists of wearable motion sensors, (Moff-Trai bands) and an application (Moff-Trai Check) loaded onto a tablet that can be used to measure physical function (Fig. 1). Given that the technology was not developed for the purpose of scientific research, the inter-rater reliability of its scoring for the outcome measures used in the PEERS trial were confirmed before the start of the trial. For this evaluation, participants aged 65 years and older were recruited from a group home in Kawasaki city (Kanagawa, Japan). Testing was conducted at the group home in August 2020 for 20 residents who consented to participate in the study.

The outcome measures used to evaluate the interventions in the PEERS trial included the 30-s chair-stand test (CS-30), the Timed Up and Go test (TUG), and the standing on one leg with eyes open (SOLEO) test. Outcomes were measured using both Moff-Trai and visual review of the recorded test videos. Moff-Trai measurements were obtained by first wrapping a Moff-Trai band around one of the participants’ thighs and launching the Moff-Trai Check application on an iPad. Thereafter, a Bluetooth connection was established between the iPad and the Moff-Trai band. Once the “start measuring” button in the application was tapped and the subject started the test, the measurements were performed automatically. One author (Y.K.) recorded the video and conducted the visual scoring. The visual scores were treated as the “true values” for this analysis. Although the Moff-Trai application displays the results (in seconds) to two decimal places, comparisons were made only up to one decimal place given that the video camera used in the trial had a frame rate of 30 fps.

### Standard procedure for CS-30 using Moff-Trai

Participants were asked to sit on a chair with arms crossed at their wrists and held against their chest. The test score reflected the number of times the participant could stand up and sit down in 30 s [18]. Incompletely executed stands, including those not completed when the 30-s signal was given, were not counted.

### Standard procedure for TUG using Moff-Trai

Participants started from a seated position with both feet on the floor and both hands resting on their knees or thighs. The person then stood up from the chair, walked straight forward for 3 m, and at the other side of the 3-m mark on the floor, turned 180°, walked back, and sat back down in the chair in the same way they started [19]. Traditionally, the count starts timing on the word “GO” rather than the moment the person left the chair. However, the test score of this study reflected the time between the moment the person left the chair and the moment the person sat down again due to the limitation of the ICT-device.

### Standard procedure for SOLEO test using Moff-Trai

The test score was estimated by recording how many seconds the participant could continue standing on one foot with eyes open. The time is measured from the moment the other foot left the ground to the moment it returned to the ground [20]. The limit for this test was set at 120 s.

### Statistical analyses

The inter-rater reliability of the Moff-Trai scores were tested by comparing the scores from the two measurement methods using the intraclass correlation coefficient (ICC) and the Bland–Altman plot. The ICC was estimated using a mixed-effects model with measurement methods and participants as random effects (2-way random effects single rater model). We also evaluated a fixed biases whether a difference between visually determined score and Moff-Trai score was within 2 times the CS-30 score or 2 s for the TUG and the SOLEO test. Correlation coefficient between the two measurements was also calculated.

## Prevention of frailty in the Elderly living at home using Exercise with Remote Supervision: the PEERS trial

### Participants and study design

PEERS trial was a 1:1 randomized parallel-group open-label trial in Japanese older adults residents. Residents in Kobe city who had a mobile application (https://mycondition.city.kobe.lg.jp/) or who received an information leaflet distributed at Kobe city office were candidates for this trial. Those who were interested in participating in the PEER trial sent an email to the secretariat and attended a briefing session on participation in this trial through the Zoom. Eligible criteria were 1) pre-frail/robust based on the Brief Frailty Index [21] (less than three items on the Brief Frailty Index), 2) aged 65 years or older and 3) people who can communicate online and operate mobile devices without problems. Participants determined to be at risk of falling (scoring more than 6 points on the Fall Risk Index) [22] were excluded. The clinical trial registry number was UMIN000041616 (05/09/2020).

### Randomization

After consenting to participate in the study, participants were randomly assigned to two groups with 1:1 allocation ratio, namely the Exercise-Intensive program or Light-load exercise program. Participants were randomly allocated to the two groups using stratified permuted block randomization with age, sex, and baseline CS-30 score as the stratification factors. Study group allocation was performed using computer-generated pseudo random numbers. After completing the enrollment and receiving the informed consent documentation, central randomization was performed at the University of Tokyo’s Epidemiology and Biostatistics Department in the Faculty of Medicine.

### Intervention

After the randomization, participants in both programs received a 3-month exercise intervention (four weeks per month). To test the feasibility of the device, we set the Exercise-Intensive program which includes “Active Centenarian Physical Fitness Program” [23] developed with reference to the exercise program recommended by the National Institute on Aging in the United States as a standard intervention program. The Active Centenarian Physical Fitness Program was developed as a 60-min exercise program (i.e., 15-min warm-up, 30-min main exercise, and 15-min cooldown) in Kochi City in 2002 with reference to the exercise program recommended by the National Institute on Aging in the United States. The detailed program was described elsewhere [23]. And we set up a control group with the Light-load Exercise program of watching a video on a health maintenance topic instead of Active Centenarian Physical Fitness Program. Setting up the control group as a group that do not exercise at all was difficult from a feasibility standpoint, given the low participation rates were considered. Therefore, we decided to add live group-based training sessions and a social event to both programs. In both programs, 45 min live training sessions of different intensity were held once a week on Sunday via Zoom. In the Exercise-Intensive program, live training consisted of communication (i.e., cheering and discussion of daily life) and exercise, with a target of 4.5 metabolic equivalent of task (MET), including strength training (push-ups, sit-ups, etc.), gymnastics, aerobic exercise (squats, steps, boxercise, etc.). In the Light-load exercise program, live training consisted of communication (i.e., cheering and discussion of daily life) and exercise, with a target of 2.5 METs, including body stretching/yoga. The weekly schedules for the Exercise-Intensive program or Light-load exercise program are summarized in Table 1. In addition to the weekly program, twice a month a social event was held for participants, and a physical therapist remotely offered the study participants advice regarding exercise and counseling on health issues once a month in both programs.

**Table 1:** Weekly training schedule for the Exercise-Intensive program or the Light-load exercise program

	**Exercise-Intensive program**	**Light-load exercise program**
Sun	Live group-based training session, 45 min (Zoom) -Different strength tasks (4.5 METs for Exercise-Intensive program and 2.5 METs for Light-load exercise program)	
Mon	Active Centenarian Physical Fitness Program, 60 min	Video on a health maintenance topic
Tue		
Wed		
Thu		
Fri	Active Centenarian Physical Fitness Program, 60 min	Video on a health maintenance topic
Sat		

### Data collection

Data collection was conducted using the Moff-Trai and Google Forms. Baseline data (sex, age, height, weight, Brief Frailty Index, and Fall Risk Index) were collected before the start of the program. The physical function tests (CS-30, TUG, and SOLEO) were automatically uploaded via the Moff-Trai and stored on a cloud server at baseline (the same time at the baseline data) and after exercise sessions once a month (the last Sunday). Similarly, using Moff-Trai, protocol compliance (attendance to Active Centenarian Physical Fitness Program or Video on a health maintenance topic) was collected. Subjective data about the effects of the program based on questionnaire (physical strength, lower back/knee pain, mood, social interactions, conversations with family, etc.) were collected through the Google Form after 3 months program.

### Outcomes

The primary outcome of the trial was the change in CS-30 score measured using Moff-Trai after 3 months. The CS-30 is a valid, reliable measure used in previous studies to confirm changes in physical functioning in frail people [24, 25]. The secondary outcomes included TUG and SOLEO scores measured by Moff-Trai, pre- to post-participation change in self-rated health, and changes in the daily lives of the participants after the study (exercise, physical functioning, quality of daily life, psychological well-being, interests, and community involvement) [19, 20]. Self-rated status was determined using the following 5-point scale before and after participating in the trial: 1, good; 2, moderate; 3, average; 4, not very good; or 5, poor. Twenty-six self-rated statuses regarding the changes in the daily lives were asked on four domains (A. exercise and physical functions, B. lifestyle, C. mental aspects, D. interests and community activities). Moreover, given that the study could involve some risk (e.g., risk of falling), adverse events were also evaluated.

### Sample size and statistical analyses

A sample size of 100 participants was used for the study. Based on long-term care prevention classes previously conducted by the Moff Company, the standard deviation for CS-30 scores was 4.5. In a small-scale randomized controlled trial conducted by Cadore et al. [26], the CS-30 scores increased by 3.6 times (increasing from 6.2 ± 4.1 to 9.8 ± 6.0) in the multicomponent intervention group (*n* = 11) and 0.9 times (increasing from 6.3 ± 3.4 to 5.4 ± 3.9) in the control group (*n* = 13) after 3 months. Assuming an equivalent effect, we hypothesized that the intervention group would show a four times larger difference and a standard deviation of around 5.7 for pre-to post-intervention improvement. Using a two-tailed significance level of 5% and a test power of 90%, a sample size of 44 participants per group was required. Considering the potential differences in the estimated effect size and standard deviation, our findings confirmed that a sample size of 100 participants would ensure that the analysis would have sufficient statistical power.

Baseline characteristics were presented as numbers and percentages for categorical variables and means or medians and standard deviations or ranges for continuous variables. Pearson’s chi-squared test and Student’s t-test or the Mann–Whitney U-test were used to compare two groups. Following the intention-to-treat principle, all statistical analyses were performed for the cases as allocated, and the two intervention groups were compared using mixed models for repeated measures (MMRM) to account for within-person correlations, utilizing the change in CS-30 score at each time point as the dependent variable. The allocated group, time point, the interaction effect between the allocated group and time point, and allocation adjustment factors (age, sex, baseline CS-30) were used as fixed effects of the independent variable. The Kenward–Roger adjustment was used for the degrees of freedom. For the covariance structure within an individual case (unstructured, 1^st^ order autoregressive, or compound symmetry), the structure that yielded the smallest Akaike information criterion was selected. We compared the 3-month change in CS-30 scores obtained based on the aforementioned model for the two groups. Furthermore, we calculated the least squares mean and 95% confidence interval (CI) for each group and the between-group difference at each time point. We also plotted the change over time using the means and standard deviations for the scores at each time point.

We conducted a subgroup analysis of each allocation factor [age (≥ 68 years/ < 68 years), sex (male/female), baseline CS-30 (15 points and more/less than 15 points)] related to the primary outcome using MMRM. Additionally, we also evaluated the influence of participant compliance for each program. A complier was defined as a participant who had attended 75% or more of all sessions (active centenarian physical fitness program in the Exercise-Intensive program, and video on a health maintenance topic Light-load exercise program). To estimate the complier-averaged treatment effect, defined as the average causal effect of the program on those who complies with their assignments, inverse probability of censoring weight generalized estimating equations were used after censoring data for participants if the compliance was lower than 75% [27].

The 3-month changes in the TUG and SOLEO test scores of the participants were analyzed in the same way as the primary analysis of CS-30 scores. To determine the pre- to post-intervention change in self-rated health, changes in the score between enrollment and after end of the 3-month intervention were recorded in a cross table. The prevalence rates for improvement in each group were calculated as the differences between both groups and the 95% CIs. For changes in daily life after participating in the study (exercise, physical functioning, quality of daily life, psychological well-being, interests, and community involvement), improvement prevalence rates were calculated for each item.

Statistical analyses were performed using JMP Pro 15.2.1 and SAS STAT 15.1 (SAS Institute Inc., Cary, NC, USA).

## Results

### Confirming the inter-rater reliability of the ICT device

**Fig. 2:**
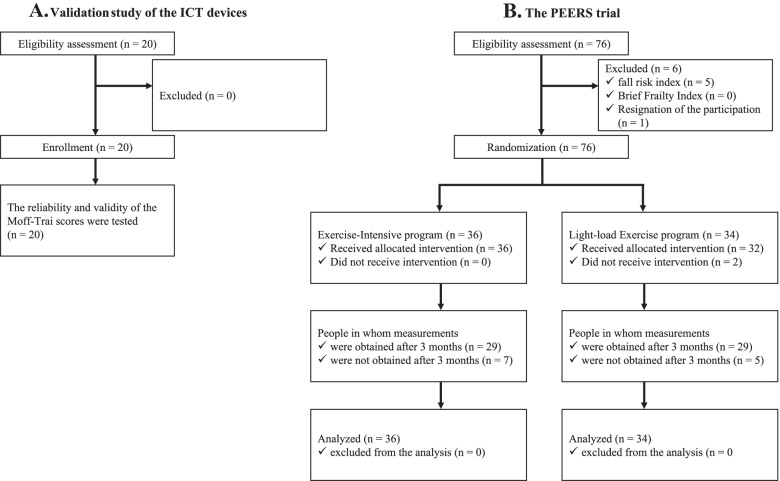
CONSORT flow diagram. **A** flow diagram enrolled in the validation study of ICT devices (Moff-Trai). **B** flow diagram enrolled in the PEERS trial. ICT, information and communications technology

**Fig. 3:**
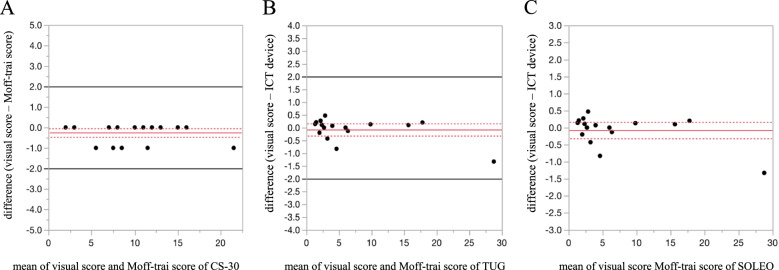
Bland–Altman plot for the CS-30, TUG, and SOLEO tests. The solid red line parallel to the *x*-axis represents the mean difference between two measurements, whereas the two dotted red lines indicate the 95% confidence interval. The solid black lines represent the permissible range for criterion-related validation. **A** 30-s chair-stand test (CS-30). **B** Time Up and Go test (TUG). **C** Standing on one leg with eyes open (SOLEO). ICT, information and communications technology

For the evaluation of the inter-rater reliability of the ICT device, all 20 participants agreed with the study (Fig. 2A). The mean age was 86.8 years [standard deviation (SD), 5.8] and 14 (70.0%) of the 20 subjects were female. The ICC values for the CS-30, TUG, and SOLEO tests were 0.995, 0.999, and 0.998, respectively. All three were over 0.99, which was close to 1. The Bland–Altman plots for the CS-30, TUG, and SOLEO tests are presented in Fig. 3. Although the upper limits for the 95% CIs of the CS-30 and the TUG were less than zero, most of the differences were distributed near zero, visually confirming that the measurement error was small. The mean differences between the scoring methods and the 95% CI for the CS-30, TUG, and SOLEO tests were − 0.25 (− 0.46, − 0.04), − 0.22 (− 0.38, − 0.07), and − 0.08 (− 0.32, 0.16, respectively. The fixed biases for the differences in measurements were within the criteria (2 times for the CS-30, and 2 s for the TUG and SOLEO tests). The correlation coefficients for the CS-30, TUG, and SOLEO tests were 0.996, 0.999, and 0.999, respectively.

## Prevention of frailty in the Elderly living at home using Exercise with Remote Supervision: the PEERS trial

### Study participants

Enrollment for this study was conducted from August to October 2020. A flow diagram based on the Consolidated Standards of Reporting Trials (CONSORT) describing the progress of the trial through the phases of enrollment, intervention allocation, follow-up, and data analysis is presented in Fig. 2B. The diagram includes the breakdown of the number of people who participated/did not participate in the intervention, the number of people in whom measurements were/were not obtained after 3 months, and the number of people included/excluded from analyses (all were included despite some missing data).

Ultimately, 70 participants were enrolled and allocated into the two groups, with the Exercise-Intensive and Light-load exercise programs comprising 36 and 34 participants, respectively. Table 2 summarizes the participants’ characteristics at baseline according to their assigned program. Accordingly, 53% of the participants were male, with a mean age of 69 years. Although both groups were found to have comparable characteristics, there was a slight difference in the distribution of the Brief Frailty Index values. Notably, 50% of participants in the Exercise-Intensive program had a score of 0, whereas only 29% of those in the Light-load exercise program had the same score. The mean baseline scores for the CS-30 were 16.83 and 18.06 in the Exercise-Intensive and Light-load exercise programs, respectively. SOLEO test scores were considerably skewed, with several participants in both groups scoring the maximum of 120 s.

**Table 2:** Baseline characteristics of participants in PEERS trial

	**Exercise-Intensive program *n* = 36**	**Light-load exercise program *n* = 34**	***P* value**
**Sex, n (%)**			0.989
**Male**	19 (52.8)	18 (52.9)	
**Female**	17 (47.2)	16 (47.1)	
**Age, years, mean (SD)**	69.3 (3.9)	68.8 (3.1)	0.568
**BMI, kg/m**^**2**^** mean (SD)**	22.2 (2.9)	23.1 (2.8)	0.182
**Brief Frailty Index, N (%)**			0.088
**0 points**	16 (44.4)	12 (35.3)	
**1 point**	9 (25.0)	16 (47.1)	
**2 points**	7 (15.4)	6 (17.7)	
**3 points**	4 (11.1)	0 (0.0)	
**Fall Risk Index N (%)**			0.244
**0 points**	18 (50.0)	10 (29.4)	
**2 points**	7 (19.4)	12 (35.3)	
**4 points**	6 (16.7)	5 (14.7)	
**5 points**	4 (11.1)	7 (20.6)	
**6 points**	1 (2.8)	0 (0.0)	
**Self-rated health status, N (%)**			0.788
**Good**	5 (18.5)	3 (10.3)	
**Moderate**	7 (25.9)	7 (24.1)	
**Average**	14 (51.9)	17 (58.6)	
**Not very good**	1 (3.7)	2 (6.9)	
**Poor**	0	0	
**CS-30, mean (SD)**	16.83 (5.34)	18.10 (4.80)	0.320
**TUG, mean (SD)**	7.59 (2.07)	7.26 (1.98)	0.508
**SOLEO, median (range)**^a^	118.6 (2.4–120.0)	60.7 (6.2–120.0)	0.051

*CS-30* 30-s chair-stand test, *TUG* Time Up and Go, *SOLEO* Standing on one leg with eyes open test, *SD* Standard deviation, *BMI* Body mass index

^a^Mann–Whitney test was used for the comparison of SOLEO

### Protocol compliance

**Fig. 4:**
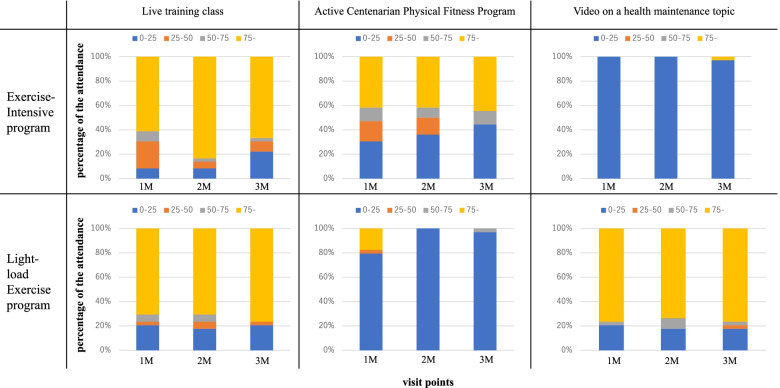
Compliance with each program for 3 months. The x-axis indicates each visit point and y-axis indicates the percentage of the attendance for each program. M, month

The monthly attendance rates for each program over 3 months are detailed in Fig. 4. For each month, participants were divided into the following 4 groups according to the percentage of the sessions that they had attended: 0%–25%, 25%–50%, 50%–75, and 75% or more.

For the live training classes, no significant difference in attendance rates was observed between the two programs. For the Active Centenarian Physical Fitness Program, approximately 40% of the participants in the Exercise-Intensive program attended more than 75% of the classes. Meanwhile, around 80% of the participants in the Light-load exercise program attended more than 75% of the video sessions. The Exercise-Intensive and Light-load exercise programs contained 11 (30.6%) and 24 (70.6%) compliers who had attended 75% or more of all sessions for each program, respectively. Although participants in the Exercise-Intensive program were not supposed to attend the video sessions and participants in the Light-load exercise program were not supposed to attend the Active Centenarian Physical Fitness Program, some participants (one participant in the Exercise-Intensive exercise program and six participants in Light-load exercise program) participated in the intervention for the other group.

### Physical function scores

**Fig. 5:**
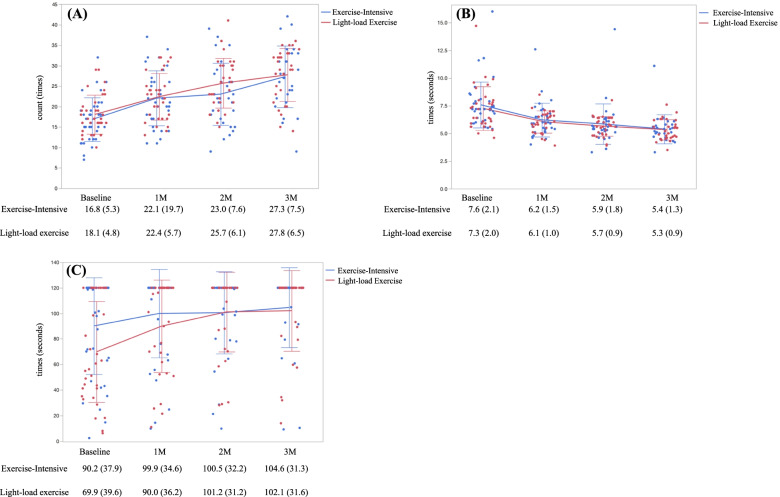
Mean changes in physical function scores for 3 months. Crude count or times of each group were plotted for each visit time. **A** 30-s chair-stand test (CS-30). **B** Time Up and Go (TUG) test. **C** Standing on one leg with eyes open (SOLEO). M, month

Figure 5 shows the crude changes in the mean (± SD) results of the CS-30, TUG, and SOLEO tests for each month.

A clear improvement in CS-30 and TUG test results was observed for both programs. Although the SOLEO test results also improved, the improvement leveled off given that the test had an upper limit of 120 s. Table 3 shows the between-group differences (Exercise-Intensive program score − Light-load exercise program score) in MMRM estimates for the least squares means of the three tests. Participants in the Exercise-Intensive and Light-load exercise programs had an estimated least square mean for the 3-month CS-30 score of 27.30 (95% CI: 25.43–29.17) and 27.22 (95% CI: 25.27–29.18), respectively. The change in the CS-30 score after 3 months was 9.89 and 9.81 following the Exercise-Intensive and Light-load exercise programs, respectively. Therefore, the primary outcome (i.e., the 3-month between-group difference in scores) was 0.08 (95% CI: − 2.64–2.79), which was not statistically significant (*p* = 0.955). Similarly, no significant differences in secondary endpoints were found (Table 3).

**Table 3:** Differences in the changes of each score after intervention

Outcomes	Program	1 month		2 months		3 months	
		**Means**^a^ **(SE)**	**Difference (95% CI)**	**Means**^a^ **(SE)**	**Difference (95% CI)**	**Means**^a^ **(SE)**	**Difference (95% CI)**
**CS-30**	**Exercise-Intensive**	22.58 (0.91)	0.88 (− 1.77, 3.54) *p* = 0.510	23.63 (0.93)	− 1.19 (− 3.88, 1.50) *p* = 0.382	27.30 (0.94)	0.08 (− 2.64, 2.79) *p* = 0.955
	**Light-load exercise**	21.69 (0.97)		24.82 (0.98)		27.22 (0.98)	
**TUG**	**Exercise-Intensive**	6.14 (0.18)	− 1.19 (− 3.88, 1.50) *p* = 0.382	5.82 (0.17)	0.10 (− 0.51, 0.70) *p* = 0.750	5.40 (0.17)	0.00 (− 0.49, 0.49) *p* = 0.989
	**Light-load exercise**	6.11 (0.19)		5.72 (0.22)		5.40 (0.17)	
**SOLEO**	**Exercise-Intensive**	94.35 (4.47)	− 1.90 (− 15.01, 11.20) *p* = 0.774	96.48 (4.58)	− 8.91 (− 22.19, 4.38) *p* = 0.187	98.99 (4.77)	− 7.68 (− 21.21, 5.84) *p* = 0.263
	**Light-load exercise**	96.26 (4.74)		105.39 (4.78)		106.67 (4.79)	

*Exercise-Intensive* Exercise-Intensive program, *Light-load exercise* Light-load exercise program, *CS-30* 30-s chair-stand test, *TUG* Timed Up and Go test, *SOLEO* Standing on one leg with eyes open test, *SE* Standard error, *CI* Confidence interval

^a^All means were least-square means after controlling covariates (baseline value, age, and sex)

Subgroup analysis based on age, sex, and baseline CS-30 scores showed that the difference in the CS-30 scores after 3 months between the Exercise-Intensive and Light-load exercise programs was consistent across the prespecified subgroups (Table 4).

**Table 4:** Subgroup analysis of differences in the change in CS-30 after 3 months

Subgroup	Program	N	LS means (SE) after 3 months	Difference (95% CI)	*P* value
**Age (< 68 years)**	**Exercise-Intensive**	14	30.47 (1.59)	1.71 (− 3.22, 6.63)	0.484
	**Light-load exercise**	13	28.77 (1.75)		
**Age (≥ 68 years)**	**Exercise-Intensive**	22	25.14 (1.07)	− 1.50 (− 4.44, 1.44)	0.310
	**Light-load exercise**	21	27.22 (1.07)		
**Male**	**Exercise-Intensive**	19	27.04 (1.38)	− 1.42 (− 5.53, 2.69)	0.490
	**Light-load exercise**	18	28.46 (1.48)		
**Female**	**Exercise-Intensive**	17	27.52 (1.32)	1.50 (− 2.32, 5.32)	0.431
	**Light-load exercise**	16	26.01 (1.34)		
**Baseline CS-30 score (< 15)**	**Exercise-Intensive**	10	20.39 (1.97)	− 1.12 (− 8.02, 5.78)	0.734
	**Light-load exercise**	9	21.51 (2.32)		
**Baseline CS-30 score (≥ 15)**	**Exercise-Intensive**	26	29.60 (1.07)	0.03 (− 3.00, 3.06)	0.986
	**Light-load exercise**	25	29.57 (1.07)		

*Exercise-Intensive* Exercise-Intensive program, *Light-load exercise* Light-load exercise program, *CS-30* 30-s chair-stand test, *LS means* Least square means, *SE* Standard error, *CI* Confidence interval

Participation compliance differed between programs (Fig. 3). The crude mean change in the CS-30 score after 3 months was 12.55 ± 6.14 among compliers (*N* = 11) and 9.39 ± 6.25 among non-compliers (*N* = 18) in the Exercise-Intensive program. Similarly, the crude mean change in the CS-30 score after 3 months was 10.65 ± 4.92 among compliers (*N* = 23) and 6.00 ± 6.35 among non-compliers (*N* = 6) in the Light-load exercise program. Compliers exhibited a greater change in the CS-30 scores after 3 months than non-compliers in both the programs (*p* = 0.014 and *p* = 0.017, respectively). However, the complier-averaged treatment effect, defined as the average causal effect of the program on those who complied with their assignments, was 1.44 (95% CI: − 2.33–5.21), without a significant difference between the programs (*p* = 0.454).

### Self-rated health

To compare the groups according to changes in self-rated health, we examined the percentage of improvement reported in each program. Accordingly, 59.2% and 51.7% of the participants in the Exercise-Intensive and Light-load exercise programs showed a decrease in their numerical ratings, respectively. Although more improvement was found in the Exercise-Intensive program, no statistical difference was observed between the programs (*p* = 0.571).

### Changes in daily life after the study

After the trial, a survey was conducted regarding changes that participants may have experienced in their daily lives. Table 5 shows the number (and percentage) of participants in each program who perceived an improvement for each item. A preferred response was obtained in questions A (5) “No more falling down” and B (5) “I have more conversations and phone calls with family and friends” in the Light-load exercise program.

**Table 5:** Questionnaire about daily life after the study

Questionnaire	Participants with improvements (%)		*P* value
	**Exercise-Intensive program**	**Light-load exercise program**	
**A. Exercise and physical functions**			
**(1) I feel physically lighter**	18 (66.7)	18 (62.1)	0.297
**(2) I feel more confident in moving my body**	19 (70.4)	19 (65.5)	0.220
**(3) I got into the habit of exercising**	21 (77.8)	21 (72.4)	0.659
**(4) Decided to continue exercising**	23 (85.2)	24 (82.8)	0.426
**(5) No more falling down**	4 (14.8)	13 (44.8)	0.037
**(6) Pain has decreased**	4 (14.8)	13 (44.8)	0.057
**B. Lifestyle**			
**(1) My daily life has become more comfortable**	13 (48.2)	15 (51.7)	0.486
**(2) My life has become more regular**	10 (37.0)	17 (58.6)	0.290
**(3) I have more opportunities and areas to go out**	4 (14.8)	7 (24.1)	0.875
**(4) I can sleep better**	9 (33.3)	10 (34.5)	0.625
**(5) I have more conversations and phone calls with family and friends**	4 (14.8)	14 (48.3)	0.021
**(6) Increased going out with family and friends**	2 (7.4)	5 (17.2)	0.212
**(7) I have more opportunities to use my smartphone or tablet**	13 (48.2)	22 (75.9)	0.102
**C. Mental aspects**			
**(1) I feel more positive**	11 (40.5)	18 (62.1)	0.166
**(2) I became more sociable**	1 (3.7)	7 (24.1)	0.090
**(3) Increased self-confidence**	7 (12.5)	12 (41.4)	0.245
**(4) I became more active**	5 (18.5)	9 (31.0)	0.521
**(5) Less anxious about falling**	6 (22.2)	14 (48.3)	0.086
**(6) Less anxious about the future**	10 (37.0)	10 (34.5)	0.428
**(7) I wanted to try new things**	18 (66.7)	23 (79.3)	0.455
**(8) I became more interested in others and the world**	9 (33.3)	16 (55.2)	0.239
**D. Interests and community activities**			
**(1) Increased the distance and time for walking**	9 (33.3)	17 (58.6)	0.178
**(2) Started (increased) hobby activities**	6 (22.2)	10 (34.5)	0.577
**(3) Began to participate in community activities (increased)**	3 (11.1)	6 (20.7)	0.300
**(4) Made new friends (increased)**	3 (11.1)	6 (20.7)	0.363
**(5) Increased e-mail and Zoom communication with family members**	6 (22.2)	11 (37.9)	0.153

### Safety

No adverse events, such as falls, occurred in either of the programs.

## Discussion

The purpose of the PEERS trial was to evaluate the feasibility of remotely delivered exercise with ICT devices in older adults living at home. “Moff-trai” showed high inter-rater reliabilities about several outcomes between visually determined score (true score) and “Moff-Trai” score. The PEERS trial showed that it could be used for remote-based training of different intensities without any safety issues. The trial revealed that the primary outcome (i.e., change in CS-30 scores after the 3-month interventions) showed a difference of only 0.08 between both programs, which was not statistically significant. Similarly, no significant differences were found for the secondary outcomes, which included changes in the TUG or SOLEO scores. Nevertheless, the least square means for the 3-month CS-30 scores were 27.30 and 27.22 for the Exercise-Intensive and Light-load exercise program, respectively, which was higher compared to the test scores obtained before participating in the trial. Notably, a meta-analysis of healthy Japanese individuals aged 60 years and older found that the standard CS-30 score for the population considered was 17.26 (95% CI: 15.98–18.55) [28], suggesting that participating in this trial improved physical functioning in both groups.

Recently, Glass et al. evaluated the association between light physical activity (between 1.6 and 2.9 METs, which was an intensity similar to that of the Light-load exercise program) and incident mobility disability in older women [29]. They not only showed that increased time spent in light-intensity physical activity was associated with reduced incident mobility disability but also that a possible threshold of the association between LPA and mobility disability exists during relatively short light-intensity physical activity (approximately 4 h per day). In this study, exercise performed once a week along with watching a video on a health maintenance topic might be sufficient to maintain/improve the short-term physical activity, considering that the participants are robust or pre-frail. These results suggest that the optimal multicomponent intervention, on which other studies have focused [8, 30], exists for community-dwelling older individuals, considering the existence of a physical activity threshold.

Another possibility could be that both programs in this trial made the participants more health-conscious, thereby increasing their daily physical activity level outside of program participation. Although this would be natural for the participants in the Exercise-Intensive program, participants in the Light-load exercise program were merely watching videos for health maintenance and having their physical functioning assessed every month, suggesting that both groups may have become more proactive than usual regarding health maintenance in their daily lives. Interestingly, compared to those participating in the Exercise-Intensive program, participants in the Light-load exercise program were more likely to have more conversations and phone calls with family and friends, to be more sociable, and to have less anxiety about falling. A previous study indicated an association between social frailty and physical frailty [31]. As such, both the Exercise-Intensive and Light-load exercise programs could have improved physical function after 3 months.

Compliers for the program showed greater improvement in physical function compared to non-compliers, although no statistically significant difference in complier-averaged treatment effects were noted between the programs. While exercise compliance has been known to affect outcomes, it is difficult to improve compliance among older people [32, 33]. Risk factors for poor compliance with the program included living alone, depression, high frailty risk score, and social risk. Deeper knowledge regarding motivation and barriers to habit changes in older people is a necessary and important line of research [34]. The PEERS trial found a higher compliance with the video on health maintenance in the Light-load exercise programs. Exchange of information received from the programs among participants may affect the daily activities and improve their physical functioning. With ICT tools, it is important to combine information regarding health maintenance to improve communication in multimodal interventions.

When the programs were compared in terms of safety, no particularly harmful incidents, such as falls, were confirmed using Moff-Trai. However, depending on the content of an exercise program, doing it at home can pose risks. Therefore, care needs to be taken when adding training exercises to a program while also confirming how safe they are when performed at home.

This study has several limitations worth noting. First, although the minimum target was 44 participants per group (88 in total), this could not be achieved because we had to close the enrollment in October 2020 (follow-up until December 2020) due to human resource issues. Thus, the current study may have lacked the statistical power to sufficiently detect significant differences. However, statistical significance would not be achieved even with the target sample size given that the observed difference was close to 0 in this study. Secondly, considering that we could not measure daily physical activity level through the device, it remained unclear how much training and other exercises participants had done, especially for the Light-load exercise program, in this study. Thirdly, this study does not evaluate the prevention of frailty (*i.e.* measures of frailty was not evaluated.) Finally, another limitation was the non-blinded assessment of the outcome measures, which increases the chance of bias for some soft endpoints.

## Conclusions

We showed a remote-based training program with ICT device in the older adults living at home was feasible. In the PEERS trials, no statistically significant differences were found between the two 3-month remote exercise interventions with differing exercise intensities. Further large-scale studies are warranted to determine what kind of remote exercise intervention programs is more effective for maintaining a physical performance and further preventing frailty.

## Data Availability

The anonymized participant data will be made available upon request to the corresponding author. Proposals will be reviewed and approved by the investigator and collaborators based on scientific merit. After the approval of a proposal, data can be shared through a secure online platform after signing a data access agreement.
